# Improving Dyspnoea Symptom Control of Patients in Palliative Care Using a Smart Patch-A Proof of Concept Study

**DOI:** 10.3389/fdgth.2021.765867

**Published:** 2021-11-29

**Authors:** Mostafa Kamal Mallick, Sarah Biser, Aathira Haridas, Vaishnavi Umesh, Olaf Tönsing, Imrana Abdullahi Yari, Malte Ollenschläger, Maria Heckel, Christoph Ostgathe, Felix Kluge, Bjoern Eskofier, Tobias Steigleder

**Affiliations:** ^1^Machine Learning and Data Analytics Lab, Department Artificial Intelligence in Biomedical Engineering (AIBE), Friedrich-Alexander-Universität Erlangen-Nürnberg (FAU), Erlangen, Germany; ^2^Department of Palliative Medicine, Comprehensive Cancer Center Erlangen-EMN, Friedrich-Alexander-Universität Erlangen-Nürnberg, Erlangen, Germany

**Keywords:** wearable devices, dyspnoea, smart patch, palliative care, digital health

## Abstract

The world of healthcare constantly aims to improve the lives of people while nurturing their health and comfort. Digital health and wearable technologies are aimed at making this possible. However, there are numerous factors that need to be addressed such as aging, disabilities, and health hazards. These factors are intensified in palliative care (PC) patients and limited hospital capacities make it challenging for health care providers (HCP) to handle the crisis. One of the most common symptoms reported by PC patients with severe conditions is dyspnoea. Monitoring devices with sufficient comfort could improve symptom control of patients with dyspnoea in PC. In this article, we discuss the proof-of-concept study to investigate a smart patch (SP), which monitors the pulmonary parameters: (a) breathing rate (BR) and inspiration to expiration ratio (I:E); markers for distress: (b) heart rate (HR) and heart rate variability (HRV), and (c) transmits real-time data securely to an adaptable user interface, primarily geared for palliative HCP but scalable to specific needs. The concept is verified by measuring and analyzing physiological signals from different electrode positions on the chest and comparing the results achieved with the gold standard Task Force Monitor (TFM).

## 1. Introduction

Symptoms related to breathing are common among palliative care (PC) patients (1), highly distressing (2), complicate treatment and discharge (3), and their prevalence is rising (4). Dyspnoea occurs in about 30% of PC cancer patients (5) and is prevalent (about 40–60%) in non-cancer patients (1, 5). The prevalence of dyspnoea may be higher in long-term care settings (6, 7) due to the growing proportion of chronic diseases such as heart rate insufficiency (8), and chronic obstructive pulmonary disease (COPD) (3) among PC patients. Also, symptoms related to breathing are increasing steadily, and thus aggressive symptom management is required in order to enhance the quality of life (4, 9, 10).

Pain, fatigue and dyspnoea are the most common symptoms faced the PC patients. Dyspnoea was rated as both distressing and severe, and was reported spontaneously by 27% of the PC patients (11). As dyspnoea is a subjective symptom, only the patient can rate its severity and the distress caused. Thus, the detection and treatment of dyspnoea rely heavily on the patient's assessment. Due to constraints in frequency and duration of visits, assessment of symptoms and their severity becomes difficult for outpatients (12, 13). Thus, it requires identification of various biomarkers, raising the need for specialized services. It is also important for patients in ambulatory settings to recognize their PC needs promptly and for patients with cognitive impairment to relate their symptoms seamlessly. Prominent biomarkers for dyspnoea comprise breathing rate (BR), breathing sounds, and their changes over time as well as the ratio of inspiration to expiration (I:E). In addition, heart rate (HR) and heart rate variability (HRV) will help to determine the degree of breathlessness and the distress it causes.

Biomarkers from physiological signals can be monitored in real-time in three different ways: using 1) contact sensors, 2) non-contact sensors, and 3) in a hybrid manner combining contact and non-contact sensors. Contact sensors require a physical connection with a stimulus to get electrical signals from the body whereas, non-contact sensors do not require a direct connection but need to be in proximity to get the signals. Non-contact based radar sensors have high precision in detecting respiration rate and can be used to replace the use of a respiratory belt (14), but radar sensors are highly sensitive to the external environment, and the movement of the patient makes it difficult to analyze the respiration rate and other biomarkers (14). Patients in ambulatory care are not in their bed all day long, however, all the relevant biomarkers need to be measured in all situations. Thus, when a patient performs activities for daily living, a radar-based non-contact system does not provide satisfactory results. Therefore, a procedure is desirable to acquire said biomarkers in a way that is non-invasive, easy to implement, and applicable in home care by laymen. The data have to be transferred securely to specialized care services in order to tailor their therapeutic approach.

BR can be measured from several sensors such as piezo-resistive sensors (15) or flex sensors (16). BR can also be derived from electrocardiogram (ECG) and photoplethysmogram (PPG) (17). Some extant researches and algorithms show that breathing parameters can be calculated from raw ECG (18, 19). But, determining I:E, one of the crucial pulmonary parameters, is not possible with the existing sensors to the best of our knowledge. Sensor fusion would enable us to integrate and compare breathing parameters obtained from impedance measurement and raw ECG to deliver reliable and precise outputs.

In our prototype, we assess BR, I:E, HR, and HRV, focusing on the trade-off between robustness and reliability against expense and burden by the innovative system. Our smart patch (SP) was implemented and tested on four healthy subjects and the performance was compared with the TFM while placing the electrodes at different positions. In the course of performance analysis of the SP, we have achieved an overall accuracy of 92.0 ± 7.4% for HR and 77.5 ± 13.2% for BR. We still need to improve our BR and I:E extraction algorithms and also miniaturize the SP in future development.

## 2. Architecture

### 2.1. Materials and Methods

**Figure 1:**
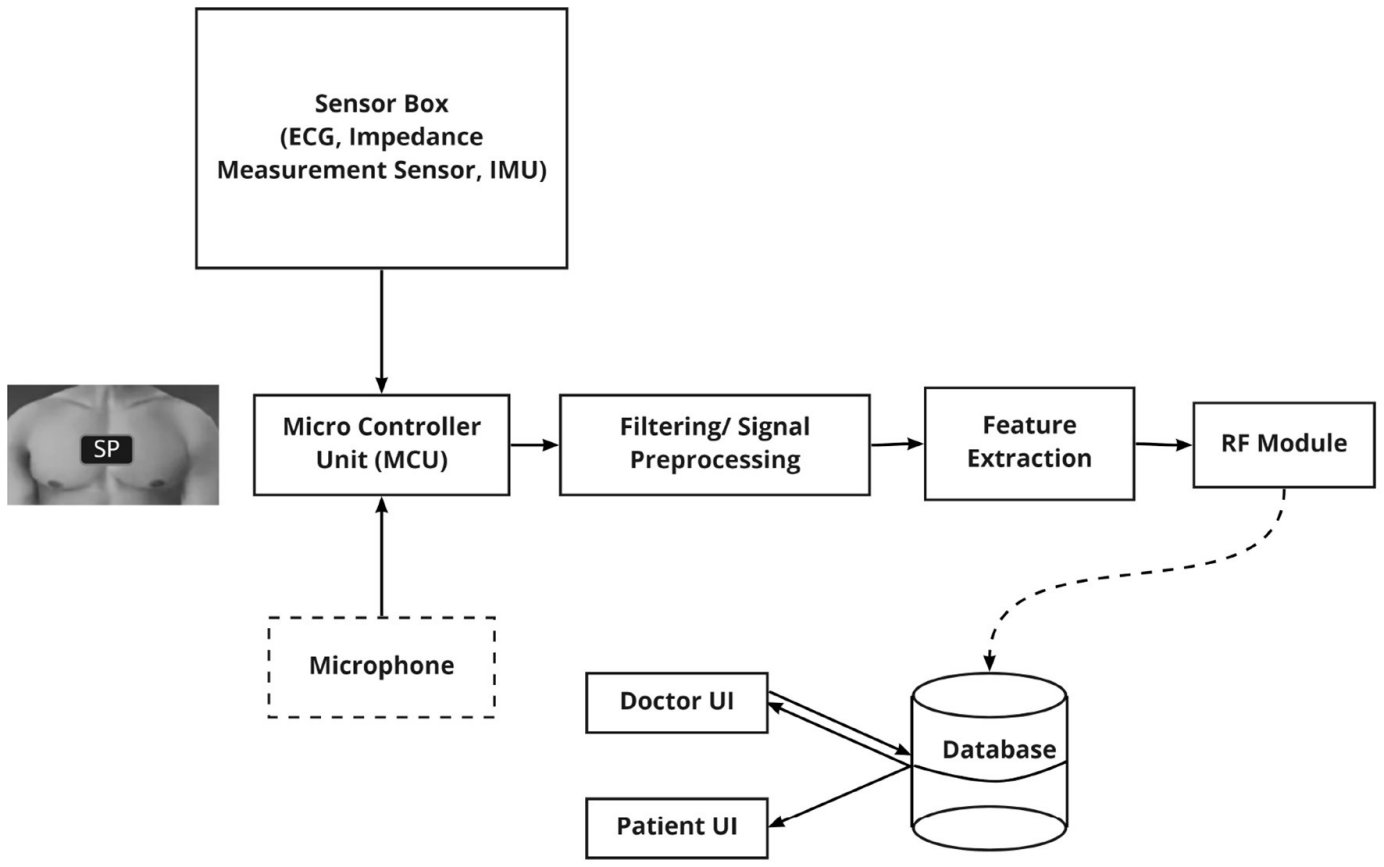
The hardware architecture of the SP: consists of a micro-controller unit (MCU) which is connected to ECG sensor, Impedance Measurement unit, Inertial Measurement Unit (IMU) and a microphone.

We have formulated our prototype as a proof-of-concept study to design and validate a SP to primarily monitor BR, I:E, HR, and HRV in real-time for PC patients. Fixed solid gel electrodes are connected to the raspberry pi with coaxial cables to measure impedance. The change of field voltage over time while breathing is measured on the person's chest to determine BR and I:E. An AD8232 SparkFun single lead heart rate monitor is employed for ECG monitoring. R- peaks of ECG were detected using the Pan Tompkins algorithm (20) and thereby HR and HRV were calculated. Further, an MPU-6000 motion interface was used as the inertial measurement unit (IMU) and an I2S SPH0645LM4H microphone was integrated with the SP prototype for future use. The overall architecture of the SP is shown schematically in Figure 1. The IMU sensor data would be used to recognize activity performed by the patient in real-time and a microphone could be used to record and store lung sounds of the patient. This information might be helpful for the HCP to diagnose and provide better care.

### 2.2. Breathing Parameters From Impedance Measurement

An impedance measurement sensor is used to independently measure the breathing parameters. However, the sensor only records impedance over time, which is why some data analysis is needed to extract parameters like BR and I:E. A fast fourier transform (FFT) was used after applying a low-pass filter to extract the dominant frequency, which is the BR. This method is found to be stable and also computationally inexpensive, but the key drawback was that it did not allow extraction of other parameters from the BR. Therefore, this is not used in the final prototype.

In our prototype, a univariate spline of fourth-order was fitted to the data after it was passed through a lowpass filter to determine BR and I:E. Since a univariate spline is just a piecewise-defined polynomial function, the first and second derivatives can be used to find minima and maxima. Then, BR is calculated from the average distance between the last five minimas, and I:E is calculated from the average distance between a minimum and the next maximum divided by the distance between a maximum to the next minimum.

### 2.3. HR and HRV From ECG

Besides BR and I:E, HR, and HRV also play an important role. The variability of HR originates from our autonomic nervous system (ANS), more concrete of different sympathetic and parasympathetic activities (21). The parasympathetic branch causes the heart to beat slower, the sympathetic one lets it beat faster, which causes a fluctuation in HR and HRV (22). The HRV can therefore be a valuable predictor in terms of distress.

The analysis of HRV involves examining the normal rhythmic fluctuations in HR using either statistical indices such as standard deviation and root mean square of the successive differences or more complex spectral analytic techniques. Since the latter is more computationally intensive and requires a lot of expertise to apply correctly, an algorithm to measure the ratio of the standard deviation of the RR-interval over the root mean square of the successive differences (rMSSD) was implemented. Balocchi et al. (23) showed that this simple statistical index could be used to surrogate the low-to-high frequency ratio, which reflects the interplay between two different branches of the ANS. The rMSSD from heart period data can be calculated by (24):


$$rMSSD=\sqrt{\frac{\sum_{i=1}^{N-1}{\left(RR_{i}-RR_{i+1}\right)}^{2}}{N-1}}\;[\text{ms}],$$

where *RR*_*i*_ is the time interval between adjacent R-waves, *RR*_*i*+1_ is the next RR-interval, and *N* is the number of RR intervals.

The HRV has a very strong clinical importance since it can predict adverse prognosis in patients with heart disease as well as in the general population. Especially in a PC setting, the interest in HRV is prominent since it indicates a risk for mortality (25). Furthermore, the BR stimulates the cardiovascular system, thereby influencing the HRV making our prototype more robust (26).

### 2.4. Psychometric Data Analysis

**Figure 2:**
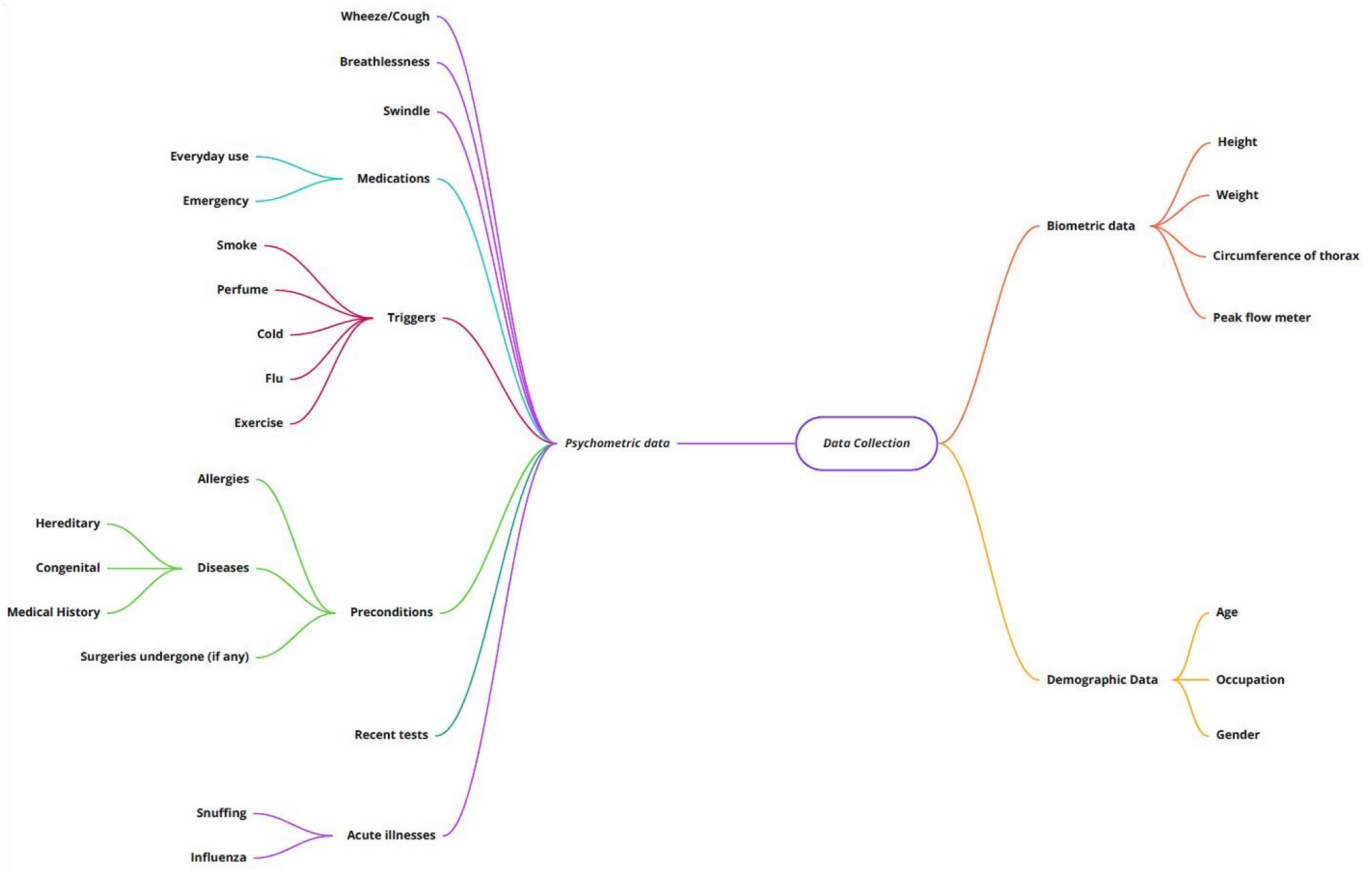
Complete overview of data to be collected.

To start the process of data collection, an overview of the necessary Psychometric data to be analyzed is key. Beyond that, an exhaustive exploration (27, 28) was performed where all possible parameters were taken into consideration for extracting the respiratory data of a patient. Figure 2 shows an overview of the different parameters.

The classification is based on biometric, demographic, and psychometric data. Basic details of the patient collected via the initial registration on the application would include how old the patient is, the gender, and the occupation because in certain situations, a COPD patient could be at the risk of asbestosis occupationally. Biometric details are used for calculating parameters like the body mass index (BMI) and certain lung volumes.

Considering psychometric data, we have a long list beginning with habitual and environmental changes like smoking, up to preconditions and medication. One of the leading causes of COPD is an alpha-1-antitrypsin deficiency which has a high prevalence among other genetic diseases along with habitual smoking. Hence, a need to consider detailed genetics arises. We included recent tests just to facilitate the patient in keeping track of their symptoms.

### 2.5. Network Architecture and Wireless Protocol

An essential part of our SP is the ability to transmit the recorded data from the raspberry pi to another machine, for example, a data server. There are several important requirements that any method of transmission must fulfill. It has to be efficient, because of the low power target that the SP will have, secure, since we are dealing with confidential patient data, and needs to be integrated into existing hospital and home infrastructure. To make sure that the data is secured during transmission, data access is handled via the secure shell (SSH) protocol. A public-private key pair was generated with the public key being stored in the raspberry pi and the private key being used to authenticate the user. Another security feature of the design is that we only pull data from the raspberry pi. Since we do not push data from the raspberry pi, man-in-the-middle attacks are almost impossible. In an eventual deployment of this system, more attention will have to be placed on security, but for testing and validation, this should be secure enough. To make sure the transmission is efficient the rsync algorithm was used.

### 2.6. Prototype Validation

To validate the aforementioned setup and the implemented algorithms, a study with four test subjects was conducted in cooperation with the PC department of the University Hospital, Erlangen. The study participants, including two males and two females, were between 24 ± 4 years of age, 176.25 ± 18.59 cm tall, 71.00 ± 6.68 kg in weight, and had no known heart or lung diseases. The recording was carried out with the participants in seated positions in a relaxed atmosphere. The purpose of the study was to compare the output of the SP to the values of the TFM. Furthermore, the study shows how close the electrodes of the SP can be placed together while getting comparable results.

**Figure 3:**
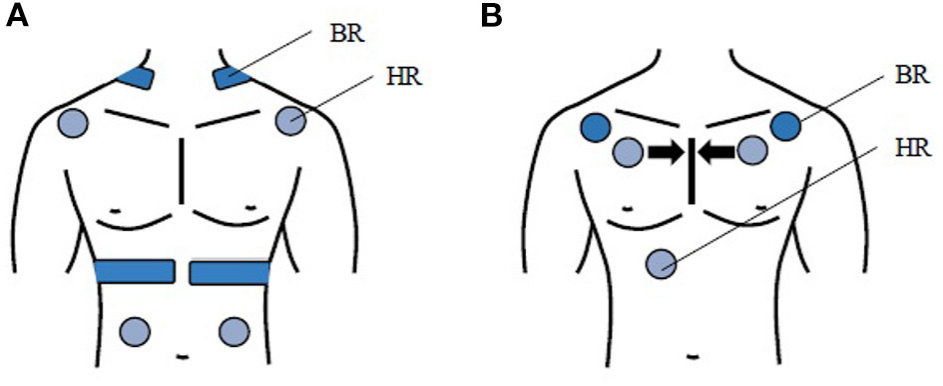
**(A)** TFM setup: measuring BR, 4 other electrodes (light blue) measuring HR, **(B)** SP setup: 2 electrodes (dark blue) measuring BR, 3 electrodes (light blue) measuring HR. During the study the electrodes of the SP are placed closer after every iteration, which is schematically shown with the black arrows.

Figure 3A shows the TFM setup: BR is measured with three electrodes (dark blue) of which two are placed on the chest and one at the back of the neck. For measuring HR, four more electrodes (light blue) are placed on the subject: two are at each side of the shoulder and two at the left and right side of the belly. Furthermore, a reference electrode is placed on the wrist of the subject. The electrodes of the TFM stay at the same place during the whole study.

Figure 3B shows the SP setup: two electrodes (dark blue) are placed on the outer part of the chest below the clavicle during the first iteration to measure BR as well as I:E. HR and HRV are measured with three further electrodes (light blue) first placed at the right mid clavicle, left mid clavicle and close to the sternum. The electrodes of the SP are placed closer together after each iteration (see arrow).

**Figure 4:**
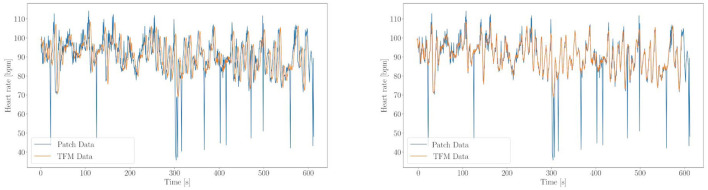
Example of timestamp matching (subject 1 position 2). On the left side HR over time is shown of SP (blue) and TFM (orange) before matching up the timestamp, on the right after. The sudden drops in the HR of the SP can be explained with missed beats (this will half the HR of the next beat since the interbeat interval is now twice as long).

For every placement, data from the TFM and SP are recorded for 10 min to be able to compare the values. The measurement of the TFM and the SP are started at the same moment. An internal delay (approximately 4 s) is observed between the first data point being recorded by TFM and the start of SP. Therefore, the timestamps are matched by correlating the recorded features manually (Figure 4). This leaves an error on the time axis of about 0.2s. Since the measurements do not start at the same time, 30 s of data are cut from the start and end of every recording. To calculate the accuracy of the SP, HR and BR are interpolated and the absolute difference is calculated between HR of the SP and TFM. This absolute difference is then integrated from 30 to 570 s, and divided by the integral of the TFM HR. This gives the relative accuracy of the SP compared to the TFM. The analysis for the BR is analogous.

## 3. Results and Discussion

**Figure 5:**
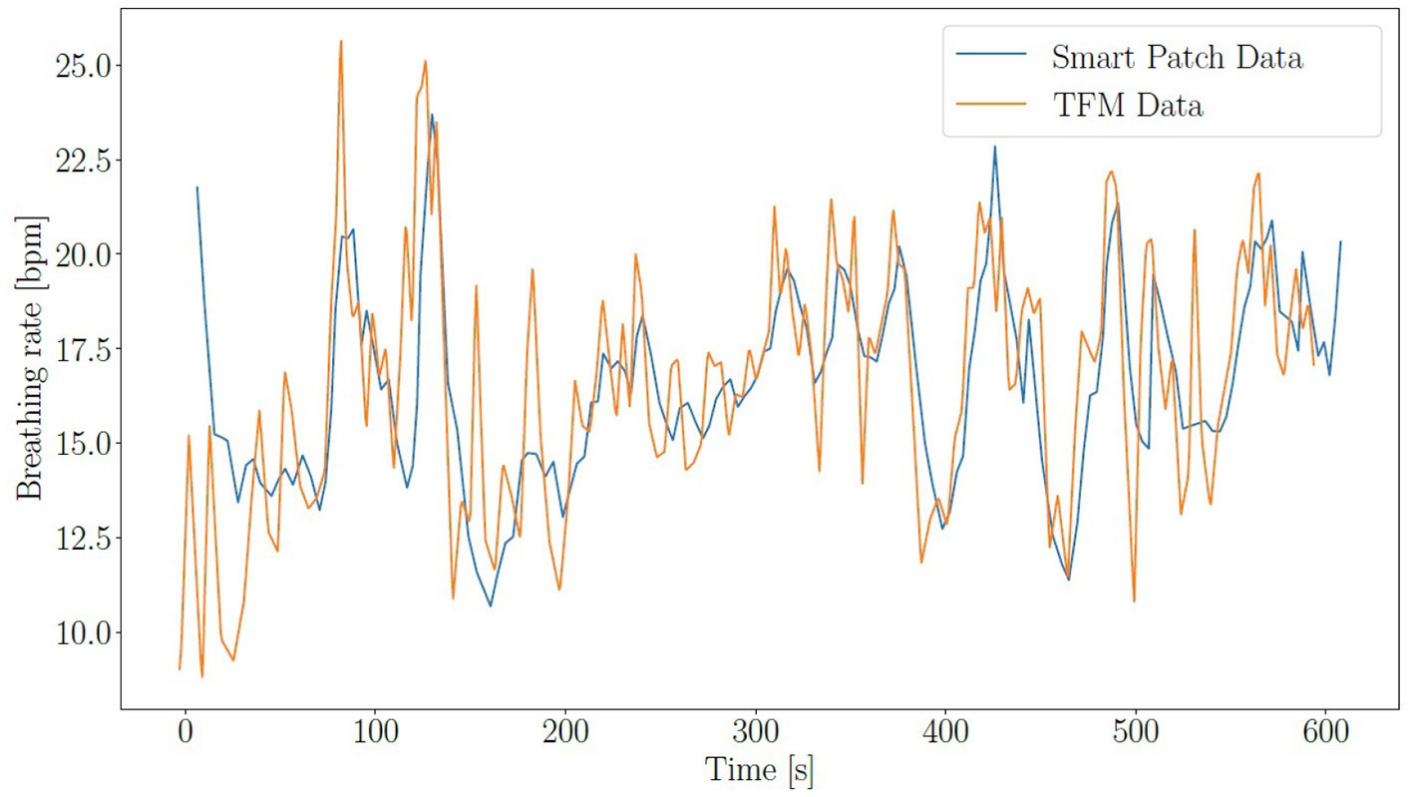
BR over time for SP (blue) and TFM (orange).

As described in subsection 2.6, the accuracy of the SP is compared with the TFM for HR and BR. The overall relative accuracy of the SP is 92.0 ± 7.4% for HR and 77.5 ± 13.2% for BR. The individual accuracy for all subjects can be found in Tables 1–4. An example for HR validation can be found in Figure 4 and for BR in Figure 5.

**Table 1:** Subject 1: Accuracy of HR and BR.

	**HR-Accuracy**	**BR-Accuracy**	**rMSSD**
Position 1	n.A.	n.A.	n.A.
Position 2	91.6%	74.7%	342ms
Position 3	97.8%	91.1%	121ms

**Table 2:** Subject 2: Accuracy of HR and BR.

	**HR-Accuracy**	**BR-Accuracy**	**rMSSD**
Position 1	98.1%	77.9%	124ms
Position 2	98.7%	83.3%	76ms
Position 3	98.6%	44.5%	96ms

**Table 3:** Subject 3: Accuracy of HR and BR.

	**HR-Accuracy**	**BR-Accuracy**	**rMSSD**
Position 1	95.0%	51.0%	217ms
Position 2	n.A.	n.A.	n.A.
Position 3	55.3%	n.A.	2264ms

**Table 4:** Subject 4: Accuracy of HR and BR.

	**HR-Accuracy**	**BR-Accuracy**	**rMSSD**
Position 1	97.0%	95.2%	155ms
Position 2	89.1%	91.4%	415ms
Position 3	91.6%	80.0%	339ms

The accuracy varies a lot between measurements, even for the same subjects while changing the electrode positions (Position 1: farthest, Position 3: closest). There are several factors causing the variation in results. One major factor could be the level of contact between the electrodes and the body. In men with a lot of body hair, the contact often varied for different positions. The impedance measurement relies heavily on good contact, thereby impacting BR in case of feeble contact. Other factors could be the movement and inherent characteristics of the subjects. Since the electrodes are attached by cables, the movement of a subject can easily influence the readings. Impedance measurement works best if there is a significant movement of the chest during each breathing cycle. Thus, it works better on people who naturally take deep and slow breaths compared to people who take shallow and fast breaths.

PPG based smartwatches show the agreement of results between 3 and 4 beats per minute (BPM) for HR, which implies an accuracy of approximately 93% and difference of 1 breath per minute i.e., 92% approximately for BR (29). Upon comparison, our SP provides accuracy in the same range depending on positions and contact of electrodes.

**Figure 6:**
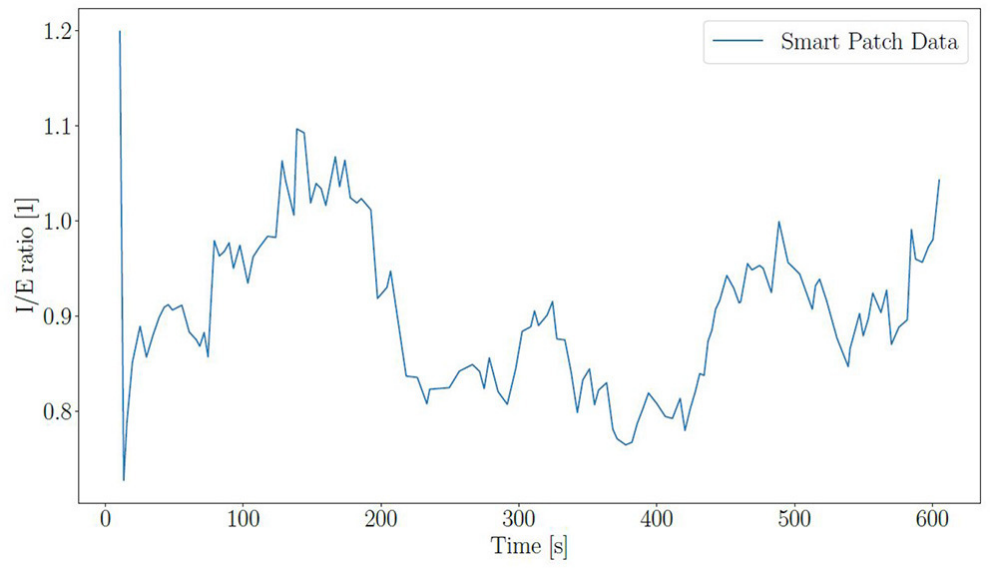
I:E for subject 1 at position 2.

Furthermore, the I:E and HRV in the form of rMSSD were also calculated by the SP. A graph of I:E over time can be found Figure 6, and the values for rMSSD are tabulated in Tables 1–4 for all the subjects. The mean value across all subjects for I:E is 1.09 ± 0.18. While this value is higher compared to the normal value of 1:2, this could be partly explained by the test situation. The subjects were aware that their breathing was being monitored and this might have changed their breathing cycle. Unfortunately, the TFM did not record I:E, thus, the corresponding SP performance could not be validated. The rMSSD is found to be higher than the normal value, which is in the range of 30ms (30). The possible reason could be missed beats which drastically increased the rMSSD. Since a missed beat means the RR-interval becoming twice as long, increasing the variance of the RR-intervals and in turn rMSSD.

## 4. Conclusion

With the population increasing at a pace faster than healthcare technologies, the necessity to monitor patients remotely, is peaking. Wearable technologies help in tackling such situations and reducing the risk of emergencies. Patients can get precise care and the HCP can understand behaviors that impact their health in real-time with wearable technologies. The feasibility of remote accumulation of physiological data and real-time analyses, increases the scope for wearable technology and in turn, improves the quality of life.

The SP developed in our study to monitor breathing parameters and other biomarkers would be an essential device for PC patients. Given the accuracy rates, we can infer that the SP has a promising future with better algorithms, incorporating proper contact and restricting artifacts. Based on this study, it is safe to conclude that the SP could be a reliable and convenient solution favoring early detection and continuous monitoring.

## 5. Future Works

In our prototype, the HR measurement shows comparable results with the TFM. But, the algorithms to determine BR and I:E need further improvement. Looking at the accuracy of our SP from section 3, it is clear that it can be reliably utilized for home monitoring after fine-tuning. Along with BR, I:E, HR, HRV, acquiring breathing sounds are also considered important biomarkers for HCP. We aspire to improve the microphone integrated into our SP to track and analyse varied breathing sounds that could be potential indicators of specific lung disorders.

In the future development, incorporating the concept of sensor fusion and determining breathing parameters such as BR and I:E from ECG along with measuring breathing parameters from the impedance measurement would make the SP more consistent, accurate, and reliable.

A predominant hindrance to wearable technologies in the field of digital health is data privacy. Any device needs to be well equipped in terms of data security and privacy as per the government rules and regulations. This is one aspect that we have to improve upon once sufficient approvals are obtained and the software application is perfected. Our final goal is to miniaturize the complete setup, conclude on a patch material and assemble the wearable SP with required software.

## Data Availability Statement

The data that support the findings of this study are available from the corresponding author upon reasonable request.

## Ethics Statement

The studies involving human participants were reviewed and approved by Ethics Committee of the Friedrich-Alexander University, Erlangen-Nuremberg. Written informed consent for participation was not required for this study in accordance with the national legislation and the institutional requirements.

## Author Contributions

IY and MO mentored the project. MH, FK, CO, and BE helped in reviewing the manuscript. TS is the ideator and medical advisor. All authors contributed to the article and approved the submitted version.

## Conflict of Interest

The authors declare that the research was conducted in the absence of any commercial or financial relationships that could be construed as a potential conflict of interest.

## Publisher's Note

All claims expressed in this article are solely those of the authors and do not necessarily represent those of their affiliated organizations, or those of the publisher, the editors and the reviewers. Any product that may be evaluated in this article, or claim that may be made by its manufacturer, is not guaranteed or endorsed by the publisher.
